# Assessment of Hypoxic Tissue Fraction and Prediction of Survival in Cervical Carcinoma by Dynamic Contrast-Enhanced MRI

**DOI:** 10.3389/fonc.2021.668916

**Published:** 2021-05-20

**Authors:** Jon-Vidar Gaustad, Einar K. Rofstad

**Affiliations:** Group of Radiation Biology and Tumor Physiology, Department of Radiation Biology, Institute for Cancer Research, Oslo University Hospital, Oslo, Norway

**Keywords:** cervical carcinoma, patient-derived xenografts, tumor hypoxia, oxygen supply, DCE-MRI

## Abstract

Tumor hypoxia is a major cause of treatment resistance and poor survival in locally-advanced cervical carcinoma (LACC). It has been suggested that *K* ^trans^ and *v*_e_ maps derived by dynamic contrast-enhanced magnetic resonance imaging can provide information on the oxygen supply and oxygen consumption of tumors, but it is not clear whether and how these maps can be combined to identify tumor hypoxia. The aim of the current study was to find the optimal strategy for calculating hypoxic fraction and predicting survival from *K* ^trans^ and *v*_e_ maps in cervical carcinoma. *K* ^trans^ and *v*_e_ maps of 98 tumors of four patient-derived xenograft models of cervical carcinoma as well as 80 patients with LACC were investigated. Hypoxic fraction calculated by using *K* ^trans^ maps correlated strongly (*P* < 0.0001) to hypoxic fraction assessed with immunohistochemistry using pimonidazole as a hypoxia marker and was associated with disease-free and overall survival in LACC patients. Maps of *v*_e_ did not provide information on hypoxic fraction and patient outcome, and combinations of *K* ^trans^ and *v*_e_ were not superior to *K* ^trans^ alone for calculating hypoxic fraction. These observations imply that *K* ^trans^ maps reflect oxygen supply and may be used to identify hypoxia and predict outcome in cervical carcinoma, whereas *v*_e_ is a poor parameter of oxygen consumption and does not provide information on tumor oxygenation status.

## Introduction

The recommended treatment for locally-advanced cervical carcinoma (LACC) is cisplatin-based chemoradiation therapy (1). Unfortunately, approximately one third of the LACC patients experience local or distant relapse after the treatment, and side effects severely reduce the quality of life for disease-free patients (2). It has been suggested that personalized treatment strategies can improve outcome for LACC patients, and the personalized strategies require prognostic biomarkers of treatment outcome (3–5). Because tumor hypoxia is a major cause of treatment resistance and poor survival in LACC patients, biomarkers reflecting the hypoxic status of tumors are warranted to personalize the treatment (4, 6, 7).

Magnetic resonance imaging (MRI) techniques, including blood oxygen level dependent MRI (BOLD-MRI), tissue oxygen level dependent MRI (TOLD-MRI), oxygen-enhanced MRI (OE-MRI), and dynamic contrast-enhanced MRI (DCE-MRI), have been applied to study tumor hypoxia (8–11). DCE-MRI is highly attractive because the technique is associated with a high signal to noise ratio and is routinely used to detect and characterize various types of cancer in the clinic. Furthermore, accumulating evidence from preclinical and clinical studies suggests that DCE-MRI can provide information on hypoxia in cervical carcinoma (12–16). A variety of strategies have been used to analyze DCE-MRI data, including semi-quantitative calculations of the signal enhancement after contrast agent administration as well as quantitative approaches using pharmacokinetic models (17). Tofs and colleagues have developed a generalized pharmacokinetic model, and this model has been shown to be preferable for analyzing human DCE-MRI data (18, 19). Tofts generalized pharmacokinetic model calculates the volume transfer rate constant (*K* ^trans^) and the fractional distribution volume of the contrast agent (*v*_e_) from contrast agent concentration *versus* time series (18). Pretreatment *K* ^trans^ and/or *v*_e_ values have been correlated with responses to radiation or chemoradiation, as well as disease-free survival (DFS) and overall survival (OS) in cervical carcinoma (20–22). However it is not clear whether and how the pharmacokinetic parameters can be used to estimate the hypoxic fraction in tumors.

Hypoxia is a result of an imbalance between oxygen supply and oxygen consumption (23). The oxygen supply is primarily determined by the blood perfusion, whereas the oxygen consumption is governed by the respiratory activity of the tissue and hence the cell density and the respiration rate of the cells (24). In our laboratory, we have investigated associations between DCE-MRI-derived parameters and hypoxia in cell line derived xenograft (CDX) models of melanoma (25, 26). In these models, we found correlations between median *K* ^trans^ and hypoxic fraction and between median *v*_e_ and hypoxic fraction (25), and we suggested that *K* ^trans^ and *v*_e_ values may be combined to calculate hypoxic fraction based on the hypothesis that *K* ^trans^ reflects oxygen supply and *v*_e_ reflects oxygen consumption (26). Recently, Hillestad et al. (27) used the same hypothesis and proposed a strategy for combining *K* ^trans^ and *v*_e_ values to determine hypoxic fractions in LACC.

Our group has also investigated associations between DCE-MRI-derived parameters and hypoxia in cervical carcinoma (12, 22, 28). In these studies, correlations were found between median *K* ^trans^ and hypoxic fraction in patient-derived xenograft (PDX) models of cervical carcinoma (12, 28), and median *K* ^trans^ was associated with DFS and OS in LACC patients (22). However, correlations between median *v*_e_ and hypoxic fraction were not found in the cervical carcinoma xenografts, and median *v*_e_ was not associated with outcome in the LACC patients (12, 22, 28). Although these studies were encouraging, the relationship between median *K* ^trans^ and hypoxic fraction was exponential rather than linear, and a strategy for calculating DCE-MRI-derived hypoxic fractions was not proposed.

The purpose of the current study was to find the optimal strategy for calculating hypoxic fractions and predicting survival from *K* ^trans^ and/or *v*_e_ maps in cervical carcinoma. We used the DCE-MRI data, the hypoxic fractions, and the DFS and OS data obtained in our previous studies (12, 22, 28), and we investigated different combinations of *K* ^trans^ and *v*_e_ to calculate hypoxic fractions and to predict patient outcome. The strategy proposed by Hillestad et al. (27) was also investigated.

## Materials and Methods

### Preclinical Data Sets

*K* ^trans^ and *v*_e_ maps as well as hypoxic fractions obtained in individual tumors of four PDX models of cervical carcinoma from two previous studies by Hauge et al. (12, 28) were used in the current study. Seventy-four data sets of untreated tumors (22 BK-12, 12 ED-15, 28 HL-16, and 12 LA-19 tumors) and 24 data sets of bevacizumab-treated tumors (10 BK-12 and 14 HL-16 tumors) were included. The DCE-MRI protocol, the immunohistochemical assay for detection of tumor hypoxia, and the bevacizumab treatment have been described in detail previously (12, 28). Briefly, BK-12, ED-15, HL-16, and LA-19 tumors were initiated in the left *quadriceps femoris* of BALB/c *nu*/*nu* mice, and were included in experiments when having grown to a volume of 100–1600 mm^3^. The PDX models have been shown to be positive for HPV E6 and E7 (29). DCE-MRI was conducted on a preclinical 7-T scanner (Bruker Biospin, Ettlingen, Germany) by using Gd-DOTA (Dotarem, Guerbet, Paris, France) as contrast agent. Dynamic T_1_-weighted images were recorded with a temporal resolution of 14.8 s and a spatial resolution of 0.23 × 0.23 × 1.0 mm^3^. Regions of interest (ROIs) encompassing the tumor tissue were delineated in T_2_-weighted anatomical images acquired prior to DCE-MRI and were transferred to the T_1_-weighted images as illustrated previously (30). Gd-DOTA concentrations were calculated by using T_1_ maps recorded before Gd-DOTA injection as detailed elsewhere (31). For each voxel, numerical values of *K* ^trans^ and *v*_e_ were calculated by using the Tofts generalized pharmacokinetic model (18) and the population-based arterial input function (*C*_a_) reported by Bejaminsen et al. (32) for BALB/c *nu*/*nu* mice.

$$C_{a}(t)=A\cdot e^{-B\cdot t}+C\cdot e^{-D\cdot t}$$

The numerical values of the constants were calculated from blood samples collected from 12 individual mice and were as follows: *A* = 2.55 mM, *B* = 0.080 s^−1^, *C* = 1.20 mM, and *D* = 0.0010 s^−1^. Plots of Gd-DOTA concentration *versus* time and the corresponding model fits for individual voxels in BK-12, ED-15, HL-16, and LA-19 tumors have been reported previously and illustrate the quality of the DCE-MRI acquisition and analysis (12). The time resolution was sufficient to produce good model fits using Tofts generalized pharmacokinetic model and the population-based arterial input function, but was too low to measure individual arterial input functions in large arteries within the field of view and for using Tofts extended pharmacokinetic model which includes the contribution from intravascular contrast agent (18, 33). Tumors were resected for histological examination immediately after DCE-MRI. Pimonidazole was used as a hypoxia marker and was administered in a single dose of 30 mg/kg 4 hours before tumor excision (34). Histological sections were prepared by standard procedures, and an anti-pimonidazole rabbit polyclonal antibody was used as primary antibody. Hypoxic fractions were assessed by image analysis and were defined as the area fraction of the viable tissue showing positive pimonidazole staining. The bevacizumab (Avastin; Hoffman-La Roche, Basel, Switzerland) treatment consisted of 3 bevacizumab doses of 10 mg/kg given over a period of 8 days.

### Clinical Data Sets

*K* ^trans^ and *v*_e_ maps as well as DFS and OS data of 80 LACC patients (FIGO stage IB through IVA) obtained by Lund et al. (22) were used in the current study. The DCE-MRI protocol, the patient characteristics, the treatment, and the follow-up have been described in detail previously (22, 35). Briefly, the size of the human tumors ranged from 2.1 to 319 cm^3^. DCE-MRI was conducted on a 1.5-T scanner (Signa; General Electric, Milwaukee, WI) by using Gd-DTPA (Schering, Berlin, Germany) as contrast agent. Dynamic T_1_-weighted images were recorded with a temporal resolution of 29 s and a spatial resolution of 0.78 × 0.78 × 5.0 mm^3^. ROIs encompassing the tumor tissue were delineated by an experienced radiologist in T_2_-weighted anatomical images acquired prior to DCE-MRI, and were transferred to T_1_-weighted and proton density images as illustrated previously (22). Gd-DTPA concentrations were calculated from T_1_-weighted and proton density images using the method of Hittmair et al. (36) as detailed elsewhere (22, 26). Numerical values of *K* ^trans^ and *v*_e_ were calculated for every voxel by using the Tofts generalized pharmacokinetic model (18) and the population-based arterial input function for humans reported by Lund et al. (22). The Lund arterial input function is similar to the arterial input function reported for mice (described above), but the numerical values of the constants differ because the uptake and clearance of small-molecular-weight contrast agents differ between mice and humans. The numerical values of the constants in the Lund arterial input function were as follows: *A* = 5.10 mM, *B* = 14.2 s^−1^, *C* = 0.99 mM, and *D* = 0.159 s^−1^ (22). Plots of Gd-DTPA concentration *versus* time and the corresponding model fits for individual voxels in the human tumors have been reported previously (22, 33), and illustrate that the quality of the DCE-MRI acquisition and analysis was sufficient to produce good model fits. After DCE-MRI, the patients were treated with concurrent cisplatin-based chemoradiation therapy with curative intent. The primary endpoints were DFS, defined as the time to local or distant relapse or death from the date of diagnosis, and OS, defined as the interval from diagnosis to death.

### Data Analysis

We investigated four strategies for calculating DCE-MRI-derived hypoxic fractions (HF_MRI_). Voxels were defined as hypoxic if $K^{trans}<K_{0}^{trans}(i),v_{e}<v_{e0}(ii),K^{trans}<K_{0}^{trans}\text{and}v_{e}<v_{e0}\;(iii)$ and $K^{trans}/K_{0}^{trans}+v_{e}/v_{e0}<1\;(iv),\text{ where }K_{0}^{trans}\text{ and}\;v_{e0}$ were threshold values for *K* ^trans^ and *v*_e_ respectively. HF_MRI_ was calculated for a range of thresholds and was compared with the hypoxic fraction assessed by immunohistochemistry (HF_PIM_) in xenografted tumors and with DFS and OS in human tumors.

In xenografted tumors, HF_MRI_ was compared with HF_PIM_ by using an adapted version of the Pearson correlation test for similarity (37):

$$Similarity\;(x,y)=\frac{\Sigma_{i=1}^{N}(x_{i}-\bar{x})(y_{i}-\bar{y})}{\frac{\left(N-1\right)}{2}(var(x)+var(y))}$$

where *x*_i_ and *y*_i_ are HF_MRI_ and HF_PIM_ of individual tumors, $\bar{x}\text{ and }\bar{y}$ are the mean values and *var*(*x*) and *var*(*y*) are the variances of HF_MRI_ and HF_PIM_ respectively, and *N* is the number of tumors. The adapted *Similarity* test was originally introduced to evaluate whether the expression of different genes showed a one-to-one correlation (37). The *Similarity* is equal to 1 if HF_MRI_ and HF_PIM_ are perfectly correlated with a slope of 1, and is lower than 1 if the correlation is imperfect or the slope differs from 1.

To compare HF_MRI_ of the human tumors with DFS and OS, the patients were divided into two groups consisting of one-third and two-third of the patients. This grouping was used because standard first-line treatment fails in approximately one-third of the patients with LACC (2). The DFS and OS of the 26 patients with the highest HF_MRI_ were compared with the DFS and OS of the 54 patients with the lowest HF_MRI_ by using the log-rank test. The comparison was performed for every threshold value to evaluate the performance of the thresholds.

### Statistical Analysis

The Pearson product moment correlation test was used to search for correlation between parameters. Curves were fitted to the data by regression analysis. Kaplan-Meier curves were compared by using the log-rank test. Probability values of *P* < 0.05 were considered significant.

## Results

### Assessment of Hypoxic Fraction in PDX Models of Cervical Carcinoma

**Figure 1:**
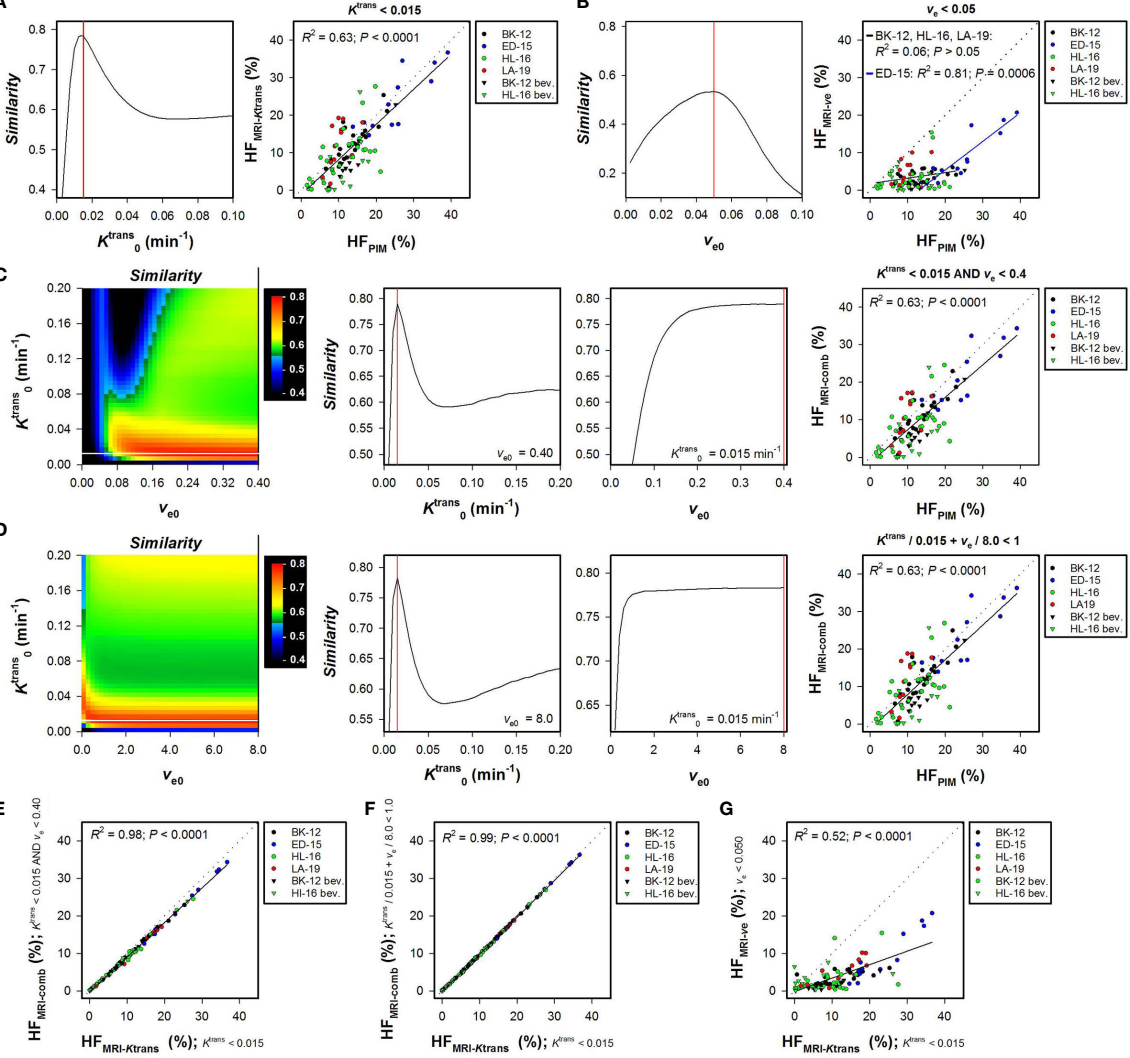
Hypoxic fractions were calculated from *K* ^trans^ maps alone (HF_MRI-_ *_K_*_trans_), *v*_e_ maps alone (HF_MRI-_ *_v_*_e_), or by combining *K* ^trans^ and *v*_e_ maps (HF_MRI-comb_), and were compared with hypoxic fractions assessed by immunohistochemistry using pimonidazole as a hypoxia marker (HF_PIM_) in untreated and bevacizumab-treated (bev) BK-12, ED-15, HL-16, and LA-19 cervical carcinoma xenografts. Voxels were defined as hypoxic if $K^{trans}<K_{0}^{trans}$ **(A)**, *v*_e_ < *v*_e0_ **(B)**, $K^{trans}<K_{0}^{trans}$ and *v*_e_ < *v*_e0_ **(C)**, or $K^{trans}/K_{0}^{trans}$ + *v*_e_/*v*_e0_ < 1 **(D)**, where $K_{0}^{trans}$ and *v*_e0_ were threshold values for *K* ^trans^ and *v*_e_ respectively. **(A)** The *Similarity* between HF_MRI-_ *_K_*_trans_ and HF_PIM_ *versus* $K_{0}^{trans}$, and HF_MRI-Ktrans_ *versus* HF_PIM_ for individual tumors using the optimal $K_{0}^{trans}$. **(B)** The *Similarity* between HF_MRI-_ *_v_*_e_ and HF_PIM_ *versus v*_e0_, and HF_MRI-_ *_v_*_e_ *versus* HF_PIM_ for individual tumors using the optimal *v*_e0_. **(C, D)** Color-coded images of the *Similarity* between HF_MRI-comp_ and HF_PIM_ for combinations of $K_{0}^{trans}$ and *v*_e0_, the *Similarity* between HF_MRI-comp_ and HF_PIM_ *versus* $K_{0}^{trans}$ for the optimal *v*_e0_, the *Similarity* between HF_MRI-comp_ and HF_PIM_ *versus v*_e0_ for the optimal $K_{0}^{trans}$, and HF_MRI-comp_ *versus* HF_PIM_ for individual tumors using the optimal combination of $K_{0}^{trans}$ and *v*_e0_. The optimal $K_{0}^{trans}$ and *v*_e0_ are indicated by white and black lines respectively in the color coded *Similarity* images, and the *Similarity* scale is given by the color bar. **(E–G)**, HF_MRI-comp_ *versus* HF_MRI-_ *_K_*_trans_ **(E, F)**, and HF_MRI-_ *_v_*_e_ *versus* HF_MRI-_ *_K_*_trans_ **(G)**. Points represent individual tumors, solid black lines were fitted to the data by linear regression analysis, and dotted lines show the one-to-one correlation line **(A–G)**. Solid red lines indicate the optimal $K_{0}^{trans}$ and the optimal *v*_e0_ **(A–D)**.

Hypoxic fractions were calculated from the *K* ^trans^ maps alone (HF_MRI-_ *_K_*_trans_), the *v*_e_ maps alone (HF_MRI-_ *_v_*_e_), or by combining the *K* ^trans^ and *v*_e_ maps (HF_MRI-comb_). **Figure 1A** shows the *Similarity* between HF_MRI-_ *_K_*_trans_ and HF_PIM_ for a range of $K_{0}^{trans}$ values, and HF_MRI-_ *_K_*_trans_ *versus* HF_PIM_ for individual tumors using the optimal $K_{0}^{trans}$. A strong one-to-one correlation was found between HF_MRI-_ *_K_*_trans_ and HF_PIM_ when the optimal $K_{0}^{trans}$ was applied (**Figure 1A**; *R* ^2^ = 0.63, *P* < 0.0001), and importantly, the same optimal $K_{0}^{trans}$ was used for untreated and bevacizumab-treated tumors of all the PDX models. **Figure 1B** shows the *Similarity* between HF_MRI-_ *_v_*_e_ and HF_PIM_ for a range of *v*_e0_ values and HF_MRI-_ *_v_*_e_ *versus* HF_PIM_ for individual tumors using the optimal *v*_e0_. The optimal *v*_e0_ resulted in HF_MRI-_ *_v_*_e_ that was lower than HF_PIM_ for most tumors, and a correlation between HF_MRI-ve_ and HF_PIM_ was only found for the ED-15 tumors implying that this strategy will have limited application (**Figure 1B**; *R* ^2^ = 0.81 and *P* = 0.0006 for ED-15 tumors; *R* ^2^ = 0.06 and *P* > 0.05 for BK-12, HL-16, and LA-19 tumors).

Two strategies were used to combine *K* ^trans^ and *v*_e_ values and calculate HF_MRI-comb_. First, voxels were defined as hypoxic if both *K* ^trans^ < $K_{0}^{trans}$ and *v*_e_ < *v*_e0_, and second, voxels were defined as hypoxic if $K^{trans}/K_{0}^{trans}$ + *v*_e_/*v*_e0_ < 1 [i.e. by applying the strategy proposed by Hillestad et al. (27)]. **Figures 1C, D** show the *Similarity* between HF_MRI-comb_ and HF_PIM_ for various combinations of $K_{0}^{trans}$ and *v*_e0_, and HF_MRI-comb_ *versus* HF_PIM_ for individual tumors by using the optimal combinations. The *Similarity* is displayed as color-coded *Similarity* images, and as graphs showing the *Similarity versus* $K_{0}^{trans}$ for the optimal *v*_e0_ and the *Similarity versus v*_e0_ for the optimal $K_{0}^{trans}$. Strong one-to-one correlations were found between HF_MRI-comb_ and HF_PIM_ for the optimal combination in both strategies (**Figures 1C, D**; *R* ^2^ = 0.63, *P* < 0.0001 for both strategies). The optimal combinations were $K_{0}^{trans}$ = 0.015 min^-1^ and *v*_e0_ = 0.40 using the first strategy (**Figure 1C**), and $K_{0}^{trans}$ = 0.015 min^-1^ and *v*_e0_ = 8.0 using the second strategy (**Figure 1D**). However, these combinations defined hypoxia essentially by considering the *K* ^trans^ value. Thus the vast majority of voxels had *v*_e_ < 0.4, and *v*_e_/8.0 was very low compared to *K* ^trans^/0.015 min^-1^ for most voxels. In line with this, strong correlations were found between HF_MRI-comb_ and HF_MRI-_ *_K_*_trans_ (**Figures 1E, F**; *R* ^2^ = 0.98 and 0.99, *P* < 0.0001 for both). A weaker correlation was found between HF_MRI-_ *_v_*_e_ and HF_MRI-_ *_K_*_trans_ (**Figure 1G**; *R* ^2^ = 0.52, *P* < 0.0001), and HF_MRI-_ *_K_*_trans_ were approximately threefold higher than HF_MRI-_ *_v_*_e_. HF_MRI-comb_ was also calculated by using the $K_{0}^{trans}$ and *v*_e0_ reported by Hillestad et al. (27), but this combination substantially overestimated the hypoxic fraction in the four PDX models used in the present study (**Supplementary Figure S1**).

**Figure 2:**
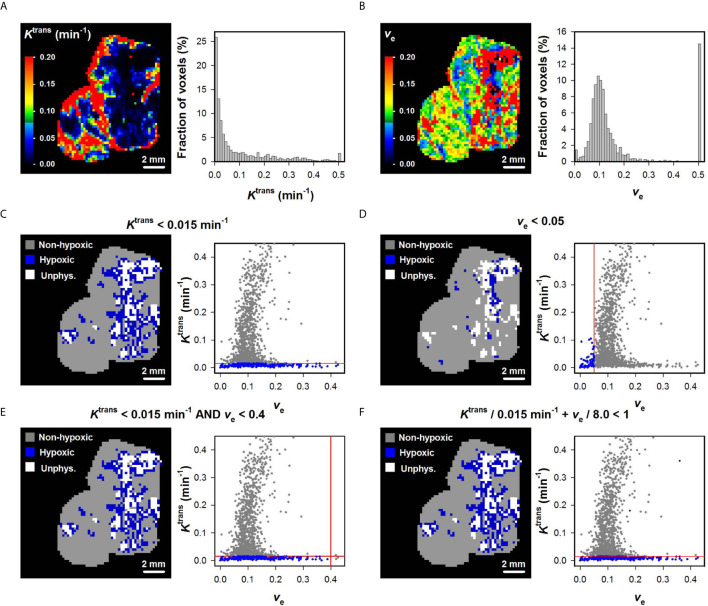
**(A, B)** *K* ^trans^ and *v*_e_ maps and histograms of a representative BK-12 cervical carcinoma xenograft. The *K* ^trans^ and *v*_e_ scales are given by the color bars. **(C–F)** DCE-MRI-derived hypoxia images and plots of *K* ^trans^ *versus v*_e_ for individual voxels of the representative BK-12 tumor. Voxels were defined as hypoxic if *K* ^trans^ < 0.015 min^-1^ **(C)**, *v*_e_ < 0.05 **(D)**, *K* ^trans^ < 0.015 min^-1^ and *v*_e_ < 0.4 **(E)**, or *K* ^trans^/0.015 min^-1^ + *v*_e_/8.0 < 1 **(F)**. Hypoxic voxels are shown in blue color, normoxic voxels are shown in gray color, and voxels with unphysiological *v*_e_ values (*v*_e_ > 1) are shown in white color. Solid red lines indicate the threshold values used to define hypoxic voxels.

The *K* ^trans^ and *v*_e_ maps and histograms of a representative BK-12 cervical carcinoma xenograft are shown in **Figures 2A, B**. **Figures 2C–F** show hypoxia images produced by applying the optimal $K_{0}^{trans}$ (**Figure 2C**), the optimal *v*_e0_ (**Figure 2D**), and the optimal combinations of $K_{0}^{trans}$ and *v*_e0_ (**Figures 2E, F**), as well as plots of *K* ^trans^ *versus v*_e_ for individual voxels of the representative BK-12 tumor. The voxels defined as hypoxic are shown in blue color in the images and plots, and the optimal discrimination line between hypoxic and normoxic voxels (i.e. the optimal $K_{0}^{trans}$ and/or *v*_e0_) are marked with red lines in the plots. The figure illustrates that the same voxels were defined as hypoxic when hypoxia was defined by the optimal $K_{0}^{trans}$ (**Figure 2C**) and when hypoxia was defined by the optimal combinations of $K_{0}^{trans}$ and *v*_e0_ (**Figures 2E, F**). When hypoxia was defined by the optimal *v*_e0_, other voxels were defined as hypoxic and the number of hypoxic voxels was substantially lower (**Figure 2D**). Voxels with unphysiological *v*_e_ values (*v*_e_ > 1) are shown in white color in the hypoxia images (**Figures 2C–F**). We have previously demonstrated that voxels consisting of necrotic tissue show a low and constant uptake of contrast agent and have unphysiological *v*_e_ values because the assumptions of the Tofts pharmacokinetic model are not fulfilled in these voxels (12, 25, 30). Interestingly, the voxels defined as hypoxic by the optimal $K_{0}^{trans}$ or by the optimal combinations of $K_{0}^{trans}$ and *v*_e0_ surrounded the voxels with unphysiological *v*_e_ values, implying that the voxels defined as hypoxic surrounded necroses as one would expect.

### Assessment of Hypoxic Fraction in LACC Patients

**Figure 3:**
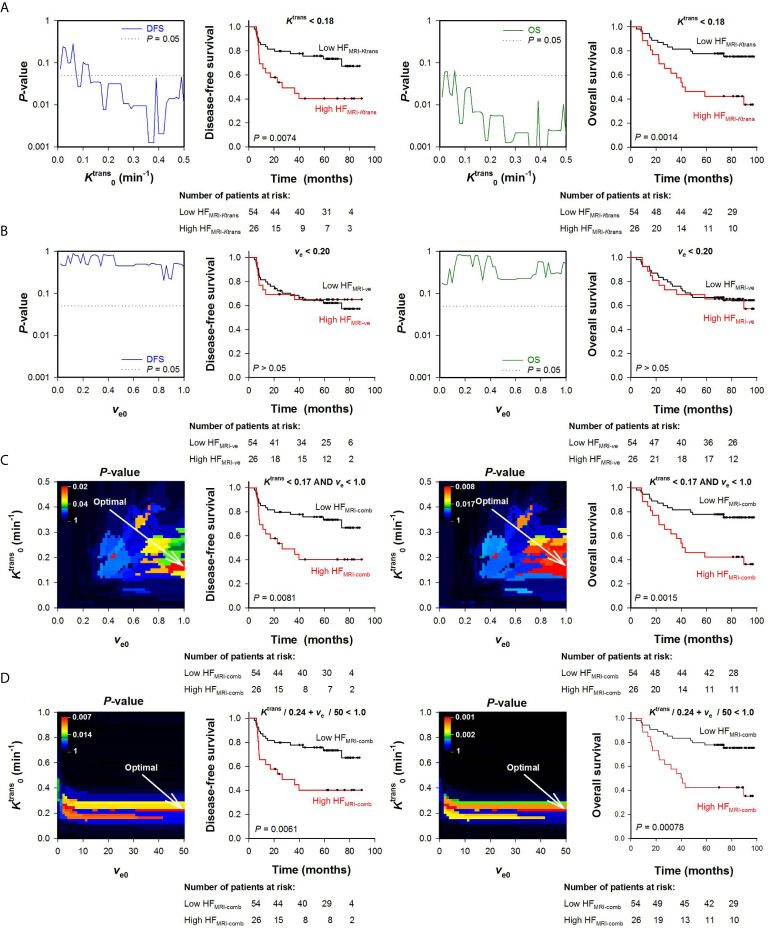
Hypoxic fractions were calculated from *K* ^trans^ maps alone (HF_MRI-_ *_K_*_trans_), *v*_e_ maps alone (HF_MRI-_ *_v_*_e_), or by combining *K* ^trans^ and *v*_e_ maps (HF_MRI-comb_) in locally-advanced cervical carcinoma patients. Voxels were defined as hypoxic if $K^{trans}<K_{0}^{trans}$ **(A)**, *v*_e_ < *v*_e0_ **(B)**, $K^{trans}<K_{0}^{trans}$ and *v*_e_ < *v*_e0_ **(C)**, or $K^{trans}/K_{0}^{trans}$ + *v*_e_/*v*_e0_ < 1 **(D)**, where $K_{0}^{trans}$ and *v*_e0_ were threshold values for *K* ^trans^ and *v*_e_ respectively. A range of $K_{0}^{trans}$ and *v*_e0_ values was investigated, and HF_MRI_ was calculated for every patient and for every threshold. The outcome of patients with high HF_MRI_ was compared with the outcome of patients with low HF_MRI_ by using the log-rank test for every threshold. **(A, B)** Plots of *P*-values obtained by the log-rank test *versus* $K_{0}^{trans}$ **(A)** or *v*_e0_ **(B)**, and examples of Kaplan-Meyer plots for patients with high and low HF_MRI-_ *_K_*_trans_ obtained by using $K_{0}^{trans}$ = 0.18 min^-1^ **(A)** or patients with high and low HF_MRI-_ *_v_*_e_ obtained by using *v*_e0_ = 0.20 **(B)**. The *P*-value plots and the Kaplan-Meyer plots refer to disease-free survival (left side) and overall survival (right side). Dotted lines in *P*-value plots show *P* = 0.05. **(C, D)** Color-coded *P*-value images obtained by the log-rank test using combinations of $K_{0}^{trans}$ and *v*_e0_, and Kaplan-Meyer plots for patients with high and low HF_MRI-conp_ obtained by using the optimal combinations of $K_{0}^{trans}$ and *v*_e0_ (indicated by arrows in the *P*-value images). The *P*-value images and the Kaplan-Meyer plots refer to disease-free survival (left side) and overall survival (right side). The numbers of patients at risk refer to the time points indicated by the time-axis of the Kaplan-Meyer plots (i.e., 0, 20, 40, 60, and 80 months). The *P*-value scales are given by the color bars.

To begin with, HF_MRI-_ *_K_*_trans_ and HF_MRI-_ *_v_*_e_ of the LACC patients was calculated from the *K* ^trans^ or the *v*_e_ maps alone. **Figure 3A** shows *P*-values obtained by the log-rank test for $K_{0}^{trans}$ values ranging from 0.005 to 0.5 min^-1^, and illustrates that a range of $K_{0}^{trans}$ values produced HF_MRI-_ *_K_*_trans_ values that were associated with outcome (*P* < 0.05). Examples of Kaplan-Meier plots for DFS and OS obtained by using a $K_{0}^{trans}$ within this range are shown in **Figure 3A**, and illustrate that the patients with high HF_MRI-_ *_K_*_trans_ had lower DFS and OS than the patients with low HF_MRI-_ *_K_*_trans_ (**Figure 3A**; DFS: *P* = 0.0074, OS: *P* = 0.0014). However, when threshold values for *v*_e_ were used to quantify HF_MRI-_ *_v_*_e_, patients with high HF_MRI-_ *_v_*_e_ did not differ from patients with low HF_MRI-_ *_v_*_e_ in survival rates (**Figure 3B**; DFS: *P* > 0.05, OS: P > 0.05 for all *v*_e0_).

**Figure 4:**
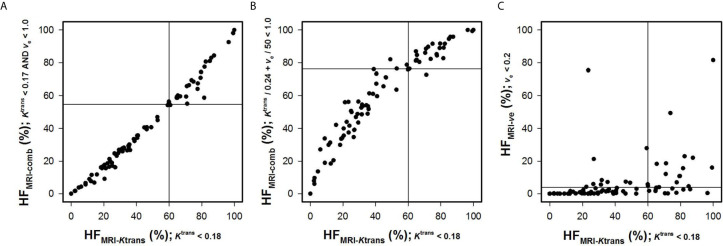
Hypoxic fractions were calculated from *K* ^trans^ maps alone (HF_MRI-_ *_K_*_trans_), *v*_e_ maps alone (HF_MRI-_ *_v_*_e_), or by combining *K* ^trans^ and *v*_e_ maps (HF_MRI-comb_) in locally-advanced cervical carcinoma patients. The plots show HF_MRI-conp_ *versus* HF_MRI-_ *_K_*_trans_ **(A, B)**, and HF_MRI-_ *_v_*_e_ *versus* HF_MRI-_ *_K_*_trans_ **(C)**. The hypoxic fractions were defined by using the thresholds (i.e. $K_{0}^{trans}$ and *v*_e0_) found in **Figure 3**. Points represent individual tumors and solid horizontal and vertical lines show the border between patients with high and low HF_MRI_.

*K* ^trans^ and *v*_e_ values were also combined to quantify HF_MRI-comb_ in the human tumors. First, voxels were defined as hypoxic if both $K^{trans}<K_{0}^{trans}$ and *v*_e_ < *v*_e0_ (**Figure 3C**), and second, voxels were defined as hypoxic if $K^{trans}/K_{0}^{trans}$ + *v*_e_/*v*_e0_ < 1 (i.e., by using the strategy proposed by Hillestad et al. (27); **Figure 3D**). **Figures 3C, D** show color-coded images of *P*-values obtained by the log-rank test for various combinations of $K_{0}^{trans}$ and *v*_e0_, and Kaplan-Meier plots for DFS and OS obtained by using the optimal combinations of $K_{0}^{trans}$ and *v*_e0_. The Kaplan-Meyer plots illustrate that the patients with high HF_MRI-comb_ had lower DFS and OS than the patients with low HF_MRI-comb_ (**Figure 3C**, DFS: *P* = 0.0081, OS: *P* = 0.0015; **Figure 3D**, DFS: *P* = 0.0061, OS: *P* = 0.0008). Interestingly, the Kaplan-Meyer plots obtained by combining *K* ^trans^ and *v*_e_ (**Figures 3C, D**) were similar to the Kaplan-Meyer plots obtained by using *K* ^trans^ alone (**Figure 3A**). Moreover, the color-coded *P*-value images illustrate that the optimal combinations of thresholds were found when a high *v*_e0_ was chosen. This implies that the hypoxic fractions were defined essentially by considering the *K* ^trans^ value. In line with this, strong correlations were found between HF_MRI-comb_ and HF_MRI-_ *_K_*_trans_ (**Figures 4A, B**; *R* ^2^ = 0.98 and 0.90, *P* < 0.0001 for both). The horizontal and vertical lines in **Figure 4** represent the border between small and large hypoxic fraction and show that the majority of the patients were stratified into the same risk group when hypoxia was defined by *K* ^trans^ alone and when hypoxia was defined by combining *K* ^trans^ and *v*_e_ (**Figures 4A, B**). No correlation was found between HF_MRI-_ *_v_*_e_ and HF_MRI-_ *_K_*_trans_ (**Figure 4C**; *R* ^2^ = 0.12, *P* > 0.05). Lastly HF_MRI-comb_ was calculated by using the $K_{0}^{trans}$ and *v*_e0_ reported by Hillestad et al. (27), but this HF_MRI-comb_ was not associated with DFS and OS in the LACC patients included in the current study (**Supplementary Figure S2**; *P* > 0.05).

## Discussion

The current study demonstrated that *K* ^trans^ maps can be used to produce hypoxia images and to calculate hypoxic fractions in cervical carcinoma. Thus a strong one-to-one correlation was found between HF_MRI-_ *_K_*_trans_ and HF_PIM_ in PDX models of cervical carcinoma, and HF_MRI-_ *_K_*_trans_ was associated with DFS and OS in LACC patients. Moreover, the same $K_{0}^{trans}$ was used to calculate HF_MRI-_ *_K_*_trans_ in untreated and bevacizumab-treated tumors of all the PDX models, whereas a range of $K_{0}^{trans}$ values was associated with outcome in the LACC patients. However, the optimal $K_{0}^{trans}$ found in the PDX models was not associated with survival in the LACC patients. This discrepancy probably reflects the vast differences in metabolism between mice and humans, and illustrates that imaging biomarkers established in xenograft models need to be verified and customized in cancer patients (5). To identify an optimal $K_{0}^{trans}$ for calculating hypoxic fraction in human LACC, novel studies that assess the hypoxic fraction by both DCE-MRI and an independent hypoxia assay are needed. Nevertheless, the associations between *K* ^trans^ derived parameters and survival rates found here may be useful also if the *K* ^trans^ derived parameters are only weakly associated with hypoxia.

According to Tofts generalized pharmacokinetic model, *K* ^trans^ is determined by the blood perfusion and the vessel permeability surface-area product (18). Preclinical studies have demonstrated that the uptake of small-molecular-weight contrast agents in tumors is limited by the blood perfusion rather than the vessel permeability (38, 39), and a recent clinical study of LACC revealed that differences in *K* ^trans^ was dominated by differences in blood perfusion and that the influence of differences in vessel permeability was negligible (40). In line with these previous studies, our findings imply that *K* ^trans^ reflected blood perfusion and oxygen supply, and that the intertumor heterogeneity in hypoxic fraction was caused by intertumor heterogeneity in blood perfusion.

Unlike *K* ^trans^, *v*_e_ did not provide information on hypoxic fraction and did not predict outcome in cervical carcinoma. Thus poor correlations were found between HF_MRI-_ *_v_*_e_ and HF_PIM_ in the PDX models, and HF_MRI-_ *_v_*_e_ was not associated with DFS and OS in LACC patients. Moreover, combinations of *K* ^trans^ and *v*_e_ were not superior to *K* ^trans^ alone for calculating hypoxic fractions, suggesting that *v*_e_ did not reflect the rate of oxygen consumption in the PDX models of cervical carcinoma and the LACC tumors.

The hypothesis that *v*_e_ reflects the oxygen consumption rate is based on two assumptions that may not be valid in cervical carcinoma. First, *v*_e_ is assumed to reflect the cell density, and second, the cell density is assumed to govern the oxygen consumption. According to Tofts generalized pharmacokinetic model, *v*_e_ is a measure of the fractional distribution volume of the contrast agent (18, 41). Small-molecular-weight contrast agents such as Gd-DTPA and Gd-DOTA are assumed to diffuse freely in the extravascular extracellular space but do not cross cell membranes and can be constrained by extracellular matrix components (18). The fractional distribution volume is thus determined by the extravascular extracellular volume fraction and the extent of extracellular matrix. If the extracellular matrix is sparse and the vascular volume is small, *v*_e_ is primarily determined by the extracellular volume fraction (ECVF) and thus directly reflects the cell density (i.e. *v*_e_ ≈ ECVF = 1 – cell density). However, several types of cancer, including cervical carcinoma, develop a dense collagen-rich extracellular matrix and display substantial intertumor heterogeneity in vessel density. In a cohort of tumors showing intertumor heterogeneity in the extent of extracellular matrix or vascular volume, *v*_e_ cannot be expected to be a good parameter of cell density. Furthermore, the rate of oxygen consumption is influenced by both the cell density and the respiration rate of the cells (24). Solid tumors have been reported to be metabolically heterogeneous and may display both intertumor and intratumor variation in cellular respiration rates (42). Values of *v*_e_ are insensitive to the cellular respiration rate, and if the cellular respiration rate varies, *v*_e_ cannot be expected to reflect the rate of oxygen consumption even in tumors where *v*_e_ reflects cell density. Taken together, these observations imply that it is unlikely that *v*_e_ can be a good parameter of the oxygen consumption rate in a large cohort of LACC patients.

We have previously found correlations between median *v*_e_ and hypoxic fraction in CDX models of melanoma (25, 26). This previous observation differs from the current finding in PDX models of cervical carcinoma, and the discrepancy probably reflects significant differences between the melanoma and cervical carcinoma models. The CDX models of melanoma showed a sparse extracellular matrix and a low vascular volume (25, 26), whereas the PDX models of cervical carcinoma develop an extensive extracellular matrix, and show substantial intertumor heterogeneity in stromal content and vessel density (29, 43). Consequently, it is likely that *v*_e_ reflected the rate of oxygen consumption and the extent of hypoxic tissue in the CDX models of melanoma but not in the PDX models of cervical carcinoma and the LACC patients.

The findings reported in the current study differ markedly from those described in a recent study by Hillestad et al. (27). In the Hillestad study, both *K* ^trans^ and *v*_e_ were associated with tumor hypoxia, and combinations of $K_{0}^{trans}$ and *v*_e0_ were reported to identify hypoxic fractions in CDX models of cervical carcinoma and LACC patients. To find the optimal combination of $K_{0}^{trans}$ and *v*_e0_ for LACC patients, Hillestad et al. (27) compared HF_MRI_ with a hypoxia gene-signature. It should be noticed that the hypoxia gene-signature was created by the same group by comparing tumors with high and low *A*_Brix_ and not by comparing tumors with high and low hypoxic fraction (44). *A*_Brix_ was calculated from DCE-MRI data by using the Brix pharmacokinetic model, and this amplitude is related to both *K* ^trans^ and *v*_e_ (18, 41). It is thus hard to agree with the authors’ claim that the *A*_Brix_-hypoxia-gene-signature is an independent measure of hypoxia, and one may suspect that the reported combination of $K_{0}^{trans}$ and *v*_e0_ was optimized to identify low *A*_Brix_-tumors rather than hypoxic tumors. Because *A*_Brix_ is related to both *K* ^trans^ and *v*_e_, it is not surprising that a combination of *K* ^trans^ and *v*_e_ provided good correlation with the *A*_Brix_-hypoxia-gene-signature. Worth to mention, the combination of $K_{0}^{trans}$ and *v*_e0_ reported by Hillestad et al. (27) was not associated with DFS or OS in our cohort of LACC patients, and was thus a poor measure of tumor hypoxia in our patient cohort.

The combination of $K_{0}^{trans}$ and *v*_e0_ that was reported to identify hypoxia in CDX models of cervical carcinoma in the Hillestad study (27) substantially overestimated the hypoxic fractions in the PDX models used here. This discrepancy may reflect differences in tumor models, but more likely reflects differences in the immunohistochemical assays used to define HF_PIM_. In our study, 30 mg/kg pimonidazole was injected 4 hours before tumor excision. This assay has been optimized to minimize unspecific staining in normoxic tissue and provides a clear distinction between tissue regions with positive and negative staining (34). Hillestad et al. (27) injected 60 mg/kg pimonidazole 90 to 120 minutes before tumor excision, and reported that their assay resulted in gradients in pimonidazole staining. Gradients in pimonidazole staining make it difficult to differentiate between normoxic and hypoxic tissue, and may have introduced uncertainties in the assessment of HF_PIM_.

Novel treatment strategies are being investigated for LACC, and some of the strategies use hypoxia-targeting therapy in combination with chemoradiation therapy (45). These strategies may be highly effective in hypoxic tumors, but the benefit of adding hypoxia-targeting therapy is more questionable in tumors with little hypoxia. The study reported here suggests that *K* ^trans^ maps derived by DCE-MRI may be used to identify LACC patients with highly hypoxic tumors that could benefit from the additional treatment.

Studies investigating the effect of antiangiogenic drugs in combination with conventional therapy have also been initiated for LACC patients (46). However, the effect of antiangiogenic drugs on tumor oxygenation is controversial because antiangiogenic treatments have been shown to normalize tumor vasculature and improve tumor oxygenation in some preclinical studies and to induce hypoxia in others (47, 48). The reason for this discrepancy is poorly understood but may have substantial impact on combination therapies (49). In the current study, strong correlations were found between HF_MRI-_ *_K_*_trans_ and HF_PIM_ in PDX models of cervical carcinoma, and the correlations were identical for untreated and bevacizumab-treated tumors. The current study thus suggests that *K* ^trans^ maps derived by DCE-MRI may be used to monitor the effect of bevacizumab treatment on tumor oxygenation in cervical carcinoma.

In summary, HF_MRI-_ *_K_*_trans_ calculated from *K* ^trans^ maps correlated with HF_PIM_ in PDX models of cervical carcinoma and was associated with DFS and OS in LACC patients, whereas maps of *v*_e_ did not provide significant information on hypoxic fraction and patient outcome. Furthermore, HF_MRI-comb_ calculated by combining *K* ^trans^ and *v*_e_ maps was not a better measure of hypoxia in cervical carcinoma than HF_MRI-_ *_K_*_trans_ calculated from *K* ^trans^ maps alone.

## Data Availability Statement

The raw data supporting the conclusions of this article will be made available by the authors, without undue reservation.

## Ethics Statement

The studies involving human participants were reviewed and approved by the regional committee of medical research ethics in southern Norway (REK sør-øst), Oslo, Norway, and was conducted in accordance with the Declaration of Helsinki. The patients/participants provided their written informed consent to participate in this study. The animal study was reviewed and approved by the Institutional Committee on Research Animal Care, Department of Comparative Medicine, Oslo University Hospital, Norway and the Norwegian Food Safety Authority (Mattilsynet), Brumunddal, Norway, and were performed in accordance with the Interdisciplinary Principles and Guidelines for the Use of Animals in Research, Marketing, and Education (New York Academy of Sciences, New York, NY, USA) and the EU Directive 2010/63/EU for animal experiments.

## Author Contributions

J-VG and ER conceived and designed the study, analyzed, and interpreted the data. J-VG wrote the manuscript. All authors contributed to the article and approved the submitted version.

## Funding

Financial support was received from the Norwegian Cancer Society and the South-Eastern Norway Regional Health Authority.

## Conflict of Interest

The authors declare that the research was conducted in the absence of any commercial or financial relationships that could be construed as a potential conflict of interest.
