# Supplementary material for: Assessment of Hypoxic Tissue Fraction and Prediction of Survival in Cervical Carcinoma by Dynamic Contrast-Enhanced MRI
Source: Front Oncol. 2021 May 20;11:668916. doi: 10.3389/fonc.2021.668916 (PMC8173130; doi:10.3389/fonc.2021.668916)
Supplement: Supplementary file 1 [file DataSheet_1.pdf]

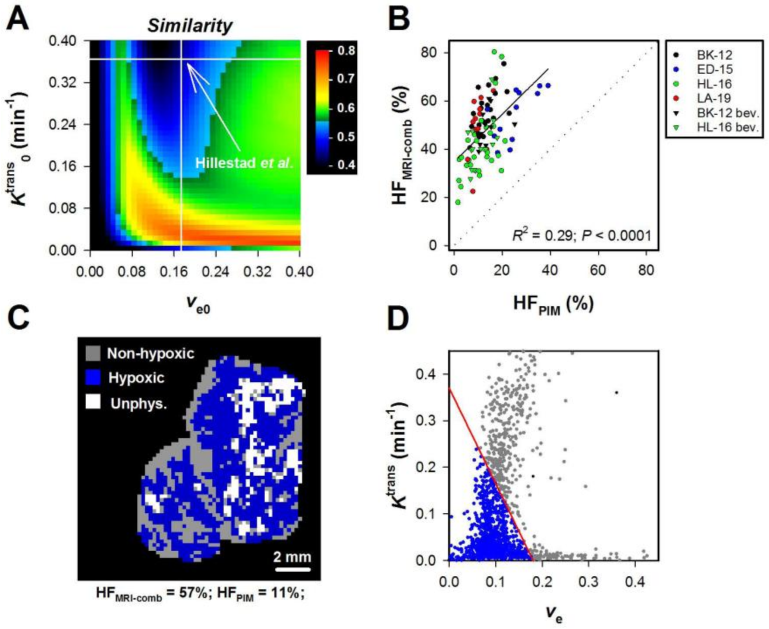

Supplementary Figure S1.

Hypoxic fractions were calculated by combining DCE-MRI derived K^{trans} and v_e maps ($HF_{MRI\text{-}comb}$) and were compared with hypoxic fractions assessed by immunohistochmistry using pimonidazole as a hypoxia marker (HF_{PIM}). Voxels were defined as hypoxic if $K^{trans} / K^{trans}_0 + v_e / v_{e0} < 1$, where K^{trans}_0 and v_{e0} were threshold values for K^{trans} and v_e respectively (i.e by using strategy proposed by Hillestad et al. (1)). **A,** Color-coded image of the *Similarity* between $HF_{MRI\text{-}comb}$ and HF_{PIM} for combinations of K^{trans}_0 and v_{e0} in untreated and bevacizumab-treated BK-12, ED-15, HL-16, and LA-19 cervical carcinoma xenografts. The *Similarity* scale is given by the color bar. The K^{trans}_0 and v_{e0} reported by Hillestad et al. (1) for HeLa and SiHa cervical carcinoma xenografts resulted in a low *Similarity* of 0.44, and are highlighted with white solid lines and a white arrow in the *Similarity* image. **B,** $HF_{MRI\text{-}comb}$ *versus* HF_{PIM} for untreated and bevacizumab-treated (bev.) BK-12, ED-15, HL-16, and LA-19 cervical carcinoma xenografts. The $HF_{MRI\text{-}comb}$ values were obtained by using the K^{trans}_0 and v_{e0} reported by Hillestad et al. (1) and were substantially higher than HF_{PIM}. Points represent individual tumors, the solid line was fitted to the data by linear regression analysis, and the dotted line shows the one-to-one correlation. **C,** DCE-MRI-derived hypoxia image of a representative BK-12 cervical carcinoma xenograft. Voxels were defined as hypoxic by using the K^{trans}_0 and v_{e0} reported by Hillestad et al. (1) and are shown in blue color, normoxic voxels are shown in gray color, and voxels with unphysiological v_e values ($v_e > 1$) are shown in white color. The K^{trans}_0 and v_{e0} reported by Hillestad et al. (1) resulted in a substantial overestimation of $HF_{MRI\text{-}comb}$ in the representative BK-12 tumor ($HF_{MRI\text{-}comb}$ = 57%, HF_{PIM} = 11%). **D,** Plot of K^{trans} *versus* v_e for individual voxels of the representative BK-12 tumor. Solid red line indicates the discrimination line between hypoxic (blue) and normoxic (gray) voxels defined by using the K^{trans}_0 and v_{e0} reported by Hillestad et al. (1).

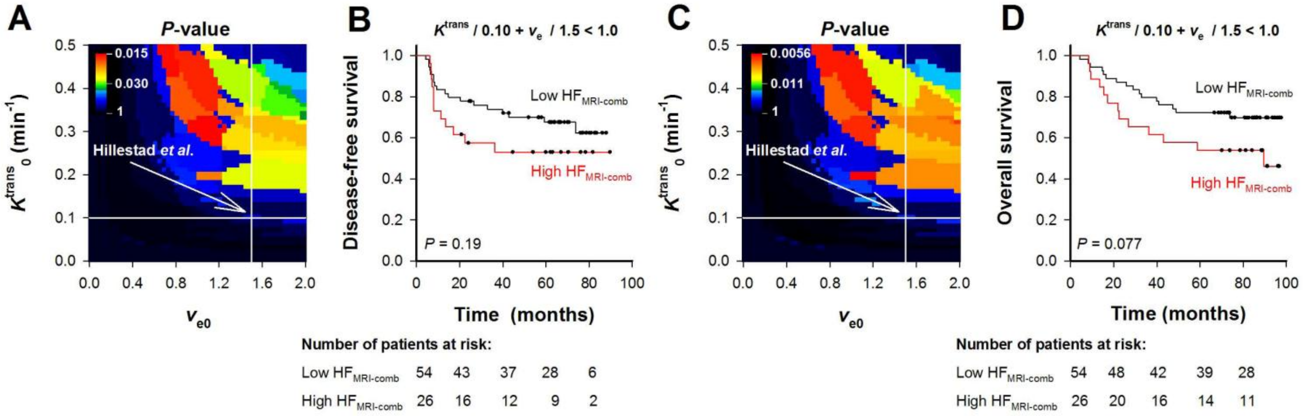

Supplementary Figure S2.

Hypoxic fractions were calculated by combining DCE-MRI derived K^{trans} and v_e maps ($HF_{MRI-comb}$) in locally-advanced cervical carcinoma (LACC) patients. Voxels were defined as hypoxic if K^{trans} / K^{trans}_0 + v_e / v_{e0} < 1, where K^{trans}_0 and v_{e0} were threshold values for K^{trans} and v_e respectively (i.e. by using the strategy proposed by Hillestad et al. (1)). The disease-free survival (DFS) and the overall survival (OS) of patients with high $HF_{MRI-comb}$ were compared with the DFS and OS of patients with low $HF_{MRI-comb}$ by using the log-rank test. **A and C,** Color-coded *P*-value images obtained by the log-rank test for DFS (A) and OS (C) using various combinations of K^{trans}_0 and $v_{e0.}$ The *P*-value scales are given by the color bars. The K^{trans}_0 and v_{e0} reported by Hillestad et al. (1) resulted in a *P*-value of 0.19 for DFS and 0.08 for OS in the cohort of LACC patients included in the current study, and are highlighted with white solid lines and a white arrow in the *P*-value images. **B and D**, Kaplan-Meyer plots for DFS (B) and OS (D) for patients with high and low $HF_{MRI-comb}$ obtained by using the K^{trans}_0 and v_{e0} reported by Hillestad et al. (1) in the cohort of LACC patients included in the current study. The numbers of patients at risk refer to the time points indicated by the time-axis of the Kaplan-Meyer plots (i.e., 0, 20, 40, 60, and 80 months).

References

1. Hillestad T, Hompland T, Fjeldbo CS, Skingen VE, Salberg UB, Aarnes EK, et al. MRI Distinguishes Tumor Hypoxia Levels of Different Prognostic and Biological Significance in Cervical Cancer. Cancer Res. 2020;80(18):3993-4003.
